# Pemafibrate prevents retinal neuronal cell death in NMDA-induced excitotoxicity via inhibition of p-c-Jun expression

**DOI:** 10.1007/s11033-020-06032-y

**Published:** 2020-12-05

**Authors:** Naoki Fujita, Kana Sase, Chihiro Tsukahara, Ibuki Arizono, Hitoshi Takagi, Yasushi Kitaoka

**Affiliations:** 1 Department of Molecular Neuroscience, St. Marianna University Graduate School of Medicine, 2-16-1 Sugao, Miyamae-ku, Kaswasaki, Kanagawa 216-8511 Japan; 2 Department of Ophthalmology, St. Marianna University School of Medicine, 2-16-1 Sugao, Miyamae-ku, Kaswasaki, Kanagawa 216-8511 Japan

**Keywords:** Excitotoxicity, PPAR-α, Pemafibrate, c-Jun, NMDA, Apoptosis

## Abstract

Excitotoxicity is involved in the retinal neuronal cell death in diabetic retinopathy. Although fenofibrate has been shown to ameliorate the progression of diabetic retinopathy, the effect of pemafibrate, which is highly selective for peroxisome proliferator-activated receptor α on retinal neuronal cell death has not been documented. Here, we investigated whether pemafibrate exerts a beneficial effect against retinal ganglion cell (RGC) death induced by N-methyl-D-aspartate (NMDA) in rats. Experiments were performed on adult male Wistar rats that received an intravitreal injection of 20 nmol NMDA. Fluoro-Gold labeled RGC morphometry showed that oral intake of pemafibrate once a day for 7 days resulted in significant protection on RGC death induced by NMDA. Phosphorylated c-Jun protein, which is involved in apoptosis, was upregulated after NMDA exposure, and this increase was significantly lessened by the systemic pemafibrate treatment. Phosphorylated c-Jun immunopositive cells were colocalized with Thy-1 immunopositive cells, and the increased these cells were ameliorated by the pemafibrate treatment. An increase in TUNEL-positive cells was significantly suppressed by the pemafibrate treatment. Phosphorylated c-Jun immunopositive cells were colocalized with TUNEL-positive cells, and they were decreased by pemafibrate treatment. These results suggest that the RGC protection achieved with pemafibrate appears to be associated with inhibition of phosphorylated c-Jun and its anti-apoptotic effect.

## Introduction

Peroxisome proliferator-activated receptors (PPARs) are involved in the body’s energy metabolism and are classified into three subtypes: PPAR-α, PPAR-β, and PPAR-γ. PPAR-α is distributed mainly in metabolic tissue, including the heart, skeletal muscle, liver, and retina, and it is activated by free fatty acids and leukotriene B4 as physiological ligands, which play a pivotal role in fatty acid metabolism [1–3]. PPAR-α has been shown to inhibit oxidative stress and degeneration in retinal neuronal cells in retinopathy model mice induced by high oxygen [4]. Fibrates are artificial ligands of PPAR-α that promote the beta-oxidation of fatty acids and lower triglycerides in the blood [5–7]. PPAR-α has recently attracted attention for its anti-inflammatory effects, but the precise mechanism by which PPAR-α confers such protection has not been elucidated. PPAR-α can inhibit the pro-inflammatory gene expression, leading to vascular inflammation reduction [5–7].

Fenofibrate is a PPAR-α agonist that has been used in the treatment of dyslipidemia. Large clinical trials, i.e., the Fenofibrate Intervention and Event Lowering in Diabetes trial [8] and the Action to Control Cardiovascular Risk in Diabetes trial [9–11], have shown that administration of fenofibrate inhibits the diabetic retinopathy progression [8–11]. A recent study has shown that fenofibrate reduces reactive oxygen species formation and ameliorates diabetic retinopathy [12]. Pemafibrate has been used as a novel treatment for fibrate dyslipidemia, and recently it has become clear that it has various functions in vivo activities [13, 14]. It has been reported to be safer than fenofibrate [15] and to be highly selective for PPAR-α [16].

c-Jun is a transcription factor belonging to the transcription factor activator protein 1 group that is phosphorylated to become p-c-Jun and is linked to the expression of apoptosis-related genes. Previous studies have reported that upregulated p-c-Jun and p-JNK are accompanied with neuronal death in the retina of diabetic rats [17], and that inhibition of p-c-Jun activity might be neuroprotective in Parkinson’s disease model rats [18].

Excess extracellular glutamate has been linked to diabetic retinopathy. For example, increase in vitreous glutamate has been shown in diabetes patients [19]. Other reports have shown that glutamate levels were increased in retinal Muller cells from diabetic rats and that such elevation might cause glutamate excitotoxicity [20, 21]. N-methyl-D-aspartate (NMDA) is a glutamate receptor agonist that is implicated in neuronal death and has been used in various studies to model neuronal cell death. Intravitreal administration of NMDA has been exhibited to activate nuclear factor-κB, inflammatory cytokines, and apoptosis-related factors and to produce retinal neurotoxicity [22]. Since diabetic retinopathy causes amacrine cell death and retinal ganglion cell (RGC) death [23–26], clarifying the mechanism of such cell death may be useful for understanding diabetic retinopathy.

In the current study, we investigated the effect of pemafibrate in rats with retinal neurotoxicity induced by NMDA and examined its alteration on p-c-Jun as a possible mechanism.

## Materials and methods

### Animals

**Fig. 1:**
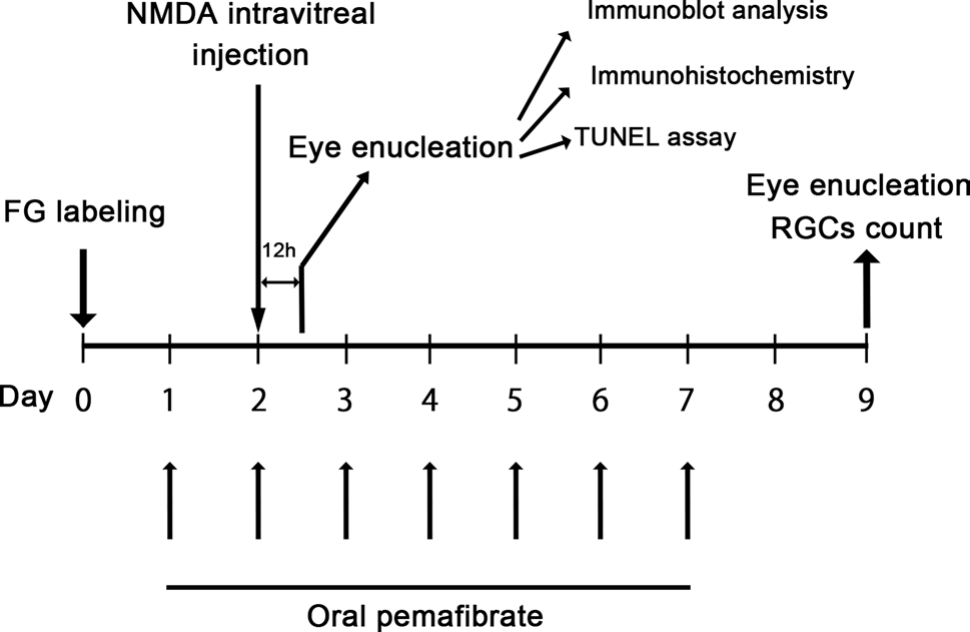
Flow diagram of the experimental procedure

A total of 41 8-week-old male Wistar rats were used for the study. The animals were housed in a room in which temperature (23 ± 1 °C), humidity (55 ± 5%), and lighting (light from 06:00 to 18:00) were controlled. Labeling of RGCs, administration of pemafibrate, intravitreal injection and measurement of RGC counts were performed on the schedule shown in Fig. 1. The study was conducted according to the Association for Research in Vision and Ophthalmology Statement for the Use of Animals in Ophthalmic and Vision Research.

### Labeling of RGCs

Rats’ RGCs were labeled retrogradely with a neurotracer, as described previously [27]. The animals were anesthetized by intramuscular injection of a mixture of ketamine and xylazine. The cerebrum was aspirated, and pieces of gelfoam soaked in a 6% solution of Fluoro-Gold (Fluorochrome, Denver, CO, USA) were placed at the superior colliculus bilaterally.

### Division of rats into groups

One day after the RGC labeling, rats were randomly assigned to administration of pemafibrate (pemafibrate group; *n* = 5) or to non-administration of pemafibrate (non-pemafibrate group; n = 5). For those in the pemafibrate group, pemafibrate 2.5 mg (10 mg/kg; provided by Kowa Co. Ltd., Nagoya, Japan) was dissolved in methylcellulose and administered orally once a day for 7 days. Non-pemafibrate group was also made.

### NMDA and PBS intravitreal administration

One day after administration/non-administration of pemafibrate, NMDA was administered intravitreally as described previously [28]. Briefly, rats were anesthetized by intraperitoneal injection of sodium pentobarbital, and 2 μl of 1 × 10^−2^ M NMDA (Sigma-Aldrich) was then injected intravitreally into the left eye of each animal under microscopy, and phosphate-buffered saline (PBS) was injected intravitreally into the rats’ right eye.

### Counting RGCs

Seven days after the intravitreal injection, the rats were killed by pentobarbital overdose, and their eyes were removed. The eyes were fixed and flat mounted. The tracer-labeled RGCs were counted at 500 μm (center area) and 2 mm (mid-periphery area) from the edge of the optic disc under a microscope (Carl Zeiss, Jena, Germany) with the use of UV filters at 8 different areas of 151,875 μm^2^ each (two areas per retinal quadrant). Distinct glial cells (bright, small, spindle-shaped cells) were excluded from the counts.

### Immunoblotting

Expression of p-c-Jun in the sensory retinas that had been removed from the enucleated eyes of 11 rats was examined by Western blotting. The retinas were homogenized in protein extraction buffer. Centrifugation was performed at 15,000 g for 15 min at 4 °C, and supernatants were obtained. The protein concentration was determined with use of a commercial protein assay kit (Bio-Rad Laboratories). Samples (8 μg each) were loaded onto SDS polyacrylamide gels (Bio-Rad Laboratories) and transferred to PVDF membranes (Immobilon-P, Merck Millipore, Tullagreen, Ireland). The membranes were incubated in Tris-buffered saline containing 0.1% Tween 20, and one of the following primary antibodies: anti-phospho-c-Jun antibody (1:200 dilution) (Cell Signaling Technology, Inc., Beverly, MA, USA) or beta-actin antibody (1:5000 dilution) (Sigma). The membranes were then reacted with peroxidase-conjugated anti-rabbit IgG or anti-mouse IgG antibody (1:5000 dilution) (Cappel, Aurora, OH, USA) and visualized with a chemiluminescence detection system (ECL Plus Western Blotting Detection Reagents). The bands were then scanned and analyzed quantitatively with the use of NIH Image software.

### Immunohistochemical analysis

Twelve hours after vitreous injection of NMDA or PBS, removed eyes of 10 rats were fixed by immersion in 4% paraformaldehyde for 24 h. After paraffin processing and sectioned, deparaffinized sections were blocked with 1% bovine serum (Roche Diagnostics GmbH, Mannheim, Germany) and incubated with anti-p-c-Jun antibody (Cell Signaling Technology, Inc.) or anti-Thy-1 antibody (Santa Cruz Biotechnology) diluted 1:100 in 1% BSA in PBS overnight. The secondary antibodies were FITC-labeled anti-rabbit antibody, or rhodamine-labeled anti-mouse antibody (Cappel, Aurora, OH, USA). Photomicrographs of the sections were obtained with a confocal microscope (LSM510, Carl Zeiss).

### TUNEL assay

TUNEL assay was used to detect retinal cells undergoing apoptosis. The assay was performed 12 h after the NMDA and PBS injections and applied in situ to 10 rats with a fluorescein apoptosis detection system (Promega, Madison, WI) [29]. TUNEL-positive cells were counted at 1.0–1.5 mm from the edge of optic disc on photos obtained with a confocal microscope (Carl Zeiss). A positive control group was also made using DNase (Promega, Madison, WI) in the PBS group.

### TUNEL combined with p-c-Jun Immunohistochemistry

After TUNEL assay, slides were double-stained with anti-p-c-Jun antibody, as described above.

### Statistical analysis

Data are presented as mean ± SEM. Differences between groups were analyzed by one-way analysis of variance (ANOVA) followed by Dunnett’s test or Mann-Whitney U test. A *P* value of less than 0.05 was considered statistically significant.

## Results

### Effects of pemafibrate on NMDA-induced neurotoxicity

**Fig. 2:**
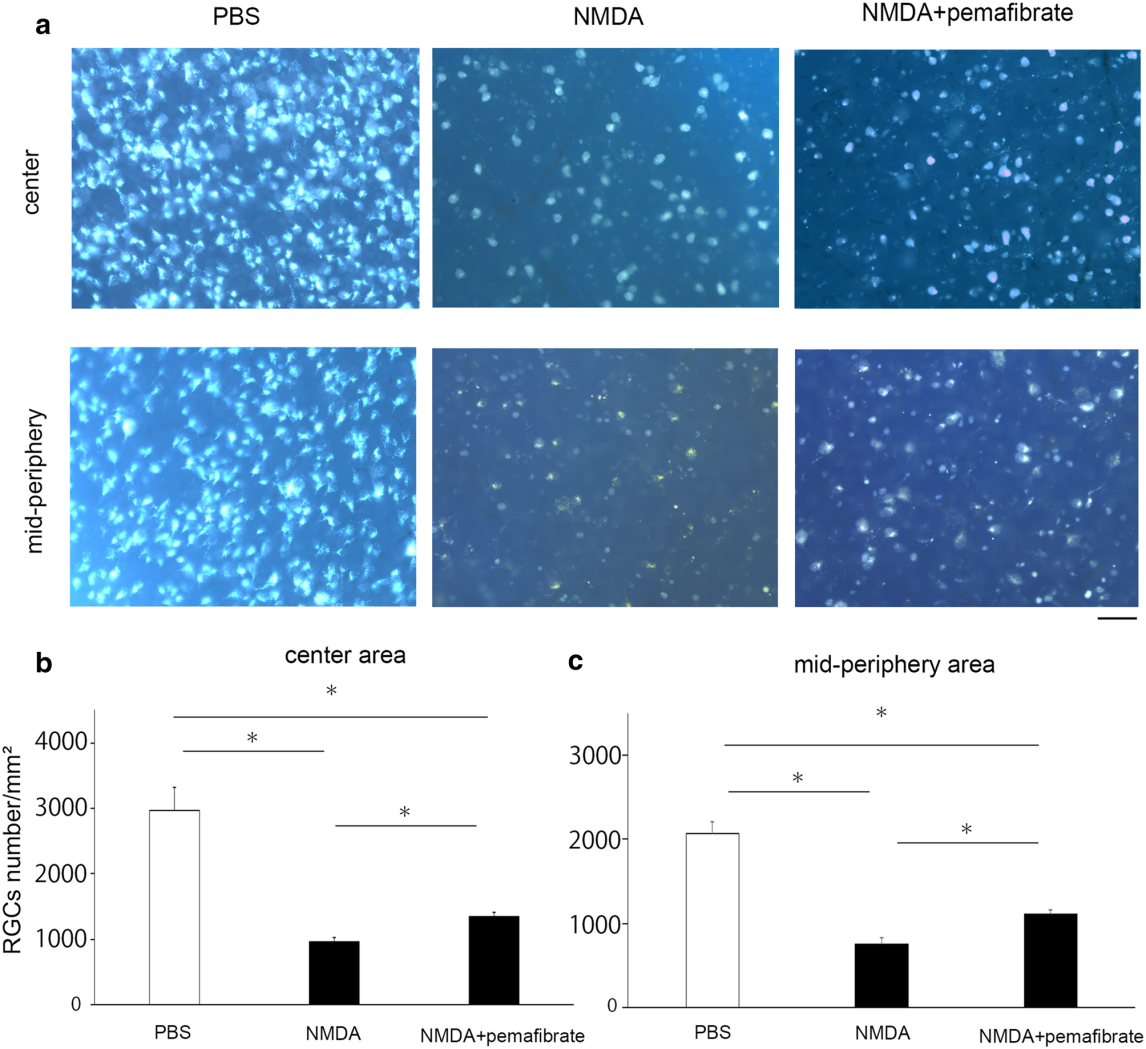
Effects of pemafibrate on RGC loss induced by NMDA. (**a**) Neurotracer-labeled RGCs in flat preparations. Scale bar = 50 μm. (**b**) Number of RGCs at center area. *n* = 4–5. *P < 0.05. (**c**) Number of RGCs at mid-periphery area. n = 4–5. *P < 0.05

Using retrograde labeling with Fluoro-Gold, we found a significant decrease in the number of RGCs in the NMDA vitreous injection group compared to the control group (Fig. 2). The number of RGCs at the center in the control group was 2963 ± 349/mm^2^, while the number was 962 ± 65/mm^2^ in the NMDA group. This decrease was significantly ameliorated in the systemic pemafibrate treatment group (RGC number was 1350 ± 61/mm^2^; *p* < 0.05; Fig. 2b). Similarly, the number of mid-periphery RGCs was 2072 ± 139/mm^2^ in the control group, while it was 752 ± 77/mm^2^ in the NMDA group (Fig. 2c). This decrease was significantly prevented by the pemafibrate treatment (RGC number was 1106 ± 48/mm^2^; *p* < 0.05; Fig. 2c).

### Effects of pemafibrate on p-c-Jun protein levels in the retina

**Fig. 3:**
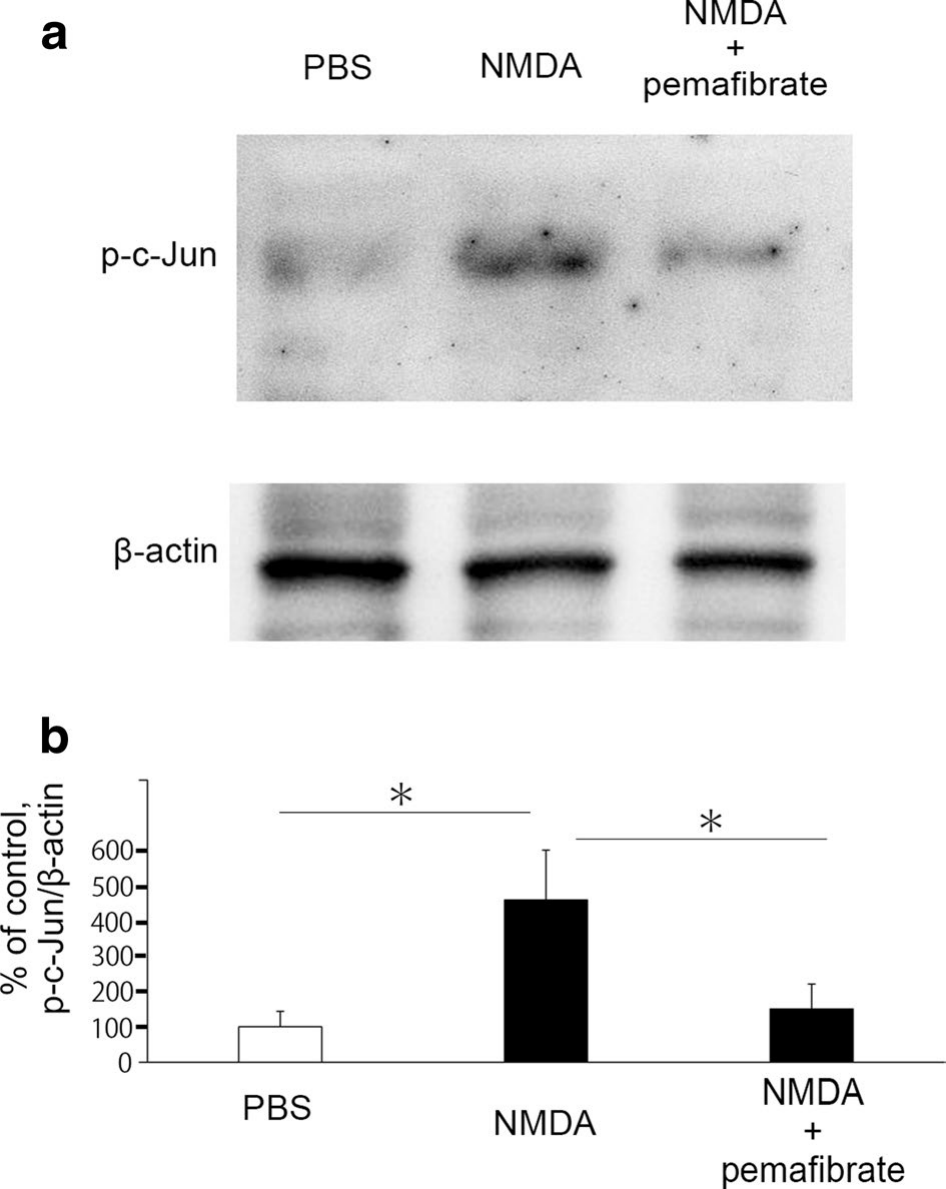
Effects of pemafibrate on p-c-Jun expression in retina. (**a**) Representative immunoblotting of p-c-Jun. (**b**) Band density. Data are normalized with β-actin. *n* = 5–6. *P < 0.05

Immunoblotting showed a significant increase in the p-c-Jun protein levels in the retina 12 h after NMDA injection compared to the control group (Fig. 3). However, this increase was significantly prevented by the systemic pemafibrate treatment (Fig. 3).

### Localization of p-c-Jun in the rat retina

**Fig. 4:**
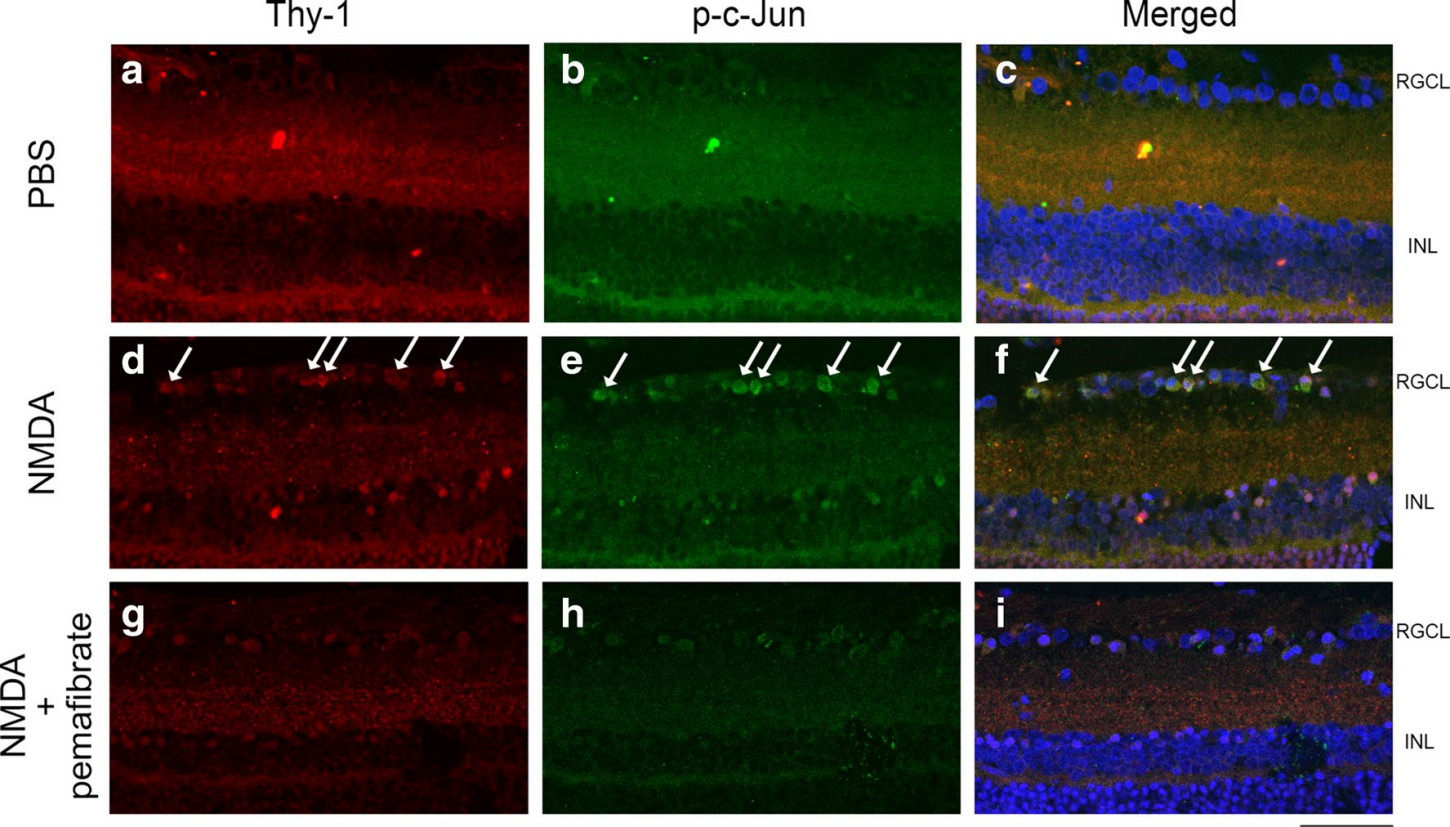
Immunohistochemistry of p-c-Jun (**b**, **e**, **h**) and Thy-1 (**a**, **d**, **g**) in retina. (**a**-**c**) PBS- treated retina, (**d**-**f**) NMDA-treated retina and (**g**-**i**) NMDA + pemafibrate treated retina. Arrows indicate colocalizations of Thy-1 and p-c-Jun immunopositive cells. Scale bar = 50 μm. *n* = 6

Immunohistochemical analysis showed that faint p-c-Jun immunoreactivity was observed in the control group, but 12 h after NMDA injury, p-c-Jun immunoreactivity was apparent (Fig. 4e). They were observed in mainly RGC layer (RGCL) and inner nuclear layer (INL) and were colocalized with Thy-1 immuno-positive cells in the RGCL (Fig. 4d-f). In the pemafibrate group, p-c-Jun immunoreactivity tended to be decreased compared to NMDA group, and they were also observed in Thy-1 positive cells in the RGCL (Fig. 4g-i).

### Effects of pemafibrate on p-c-Jun in NMDA-induced apoptosis

**Fig. 5:**
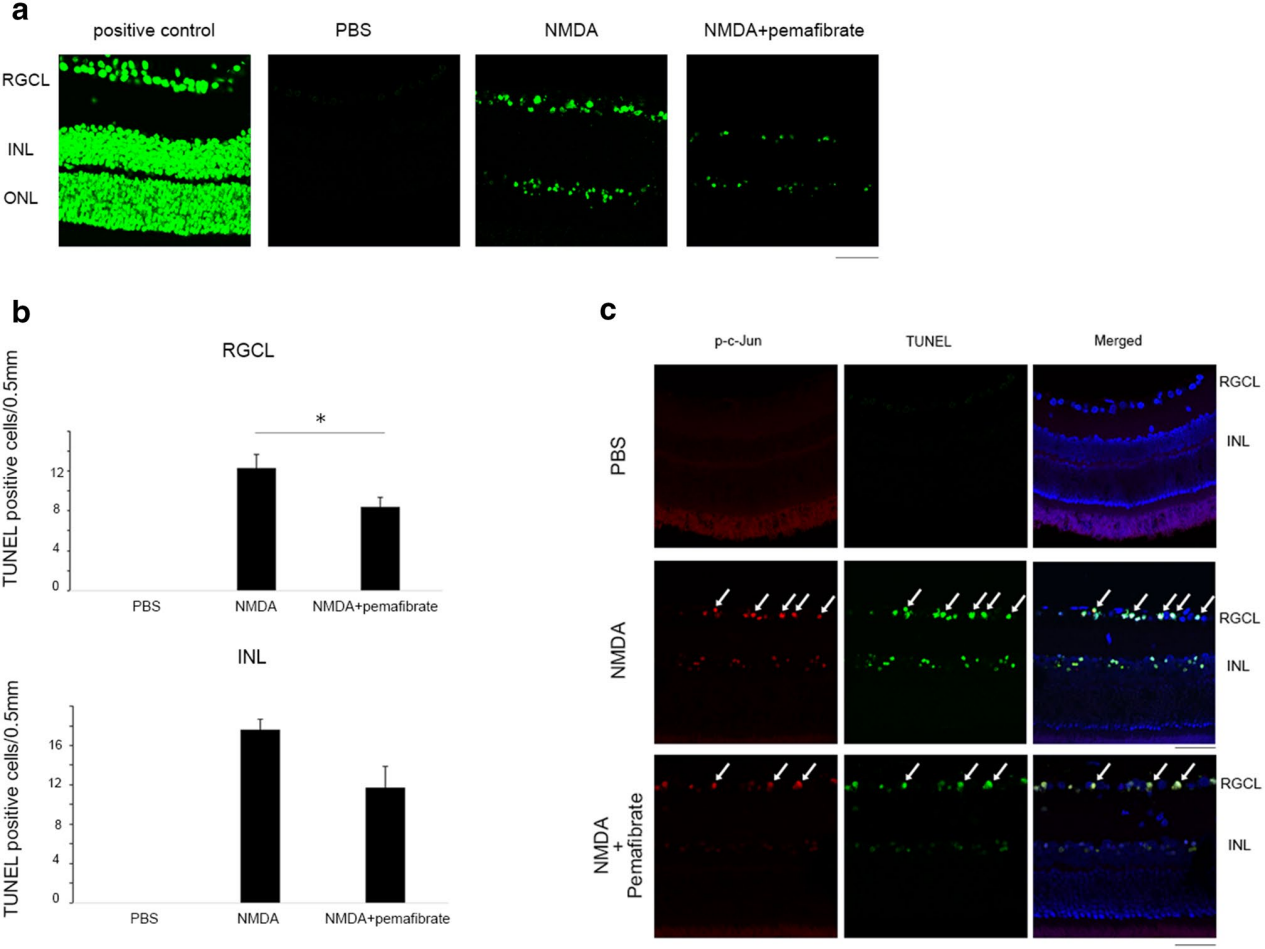
TUNEL assay in retina. (**a**) TUNEL-positive cells 12 h after NMDA injection with or without systemic pemafibrate treatment. DNase was used for positive control. TUNEL-positive cells were not seen 12 h after PBS injection. Scale bar = 50 μm. (**b**) TUNEL-positive cell number in the RGCL. *n* = 5. ^*^P < 0.05. (upper panel). TUNEL-positive cell number in the INL. n = 5. (lower panel). (**c**)Double-labeling for TUNEL and p-c-Jun immunoreactivity 12 h after NMDA injection with or without systemic pemafibrate treatment. PBS is also shown as a control. Arrows indicate colocalizations of TUNEL-positive cells and p-c-Jun immunopositive cells. Scale bar = 50 μm

TUNEL staining showed abundant apoptotic cells in the RGCL and INL in the NMDA vitreous injection group and they were decreased in the pemafibrate treatment group (Fig. 5a). The number of TUNEL-positive cells in the RGCL was significantly lower in the pemafibrate oral administration group compared to the NMDA group (Fig. 5b). Although the number of TUNEL-positive cells in the INL tended to be lower in the pemafibrate group compared to the NMDA group, the difference was not statistically significant (*p* = 0.075; Fig. 5b). Moreover, the double staining revealed that p-c-Jun immuno-positive cells were co-localized with TUNEL-positive cells after NMDA injection, indicating that they are consistent with apoptotic cells (Fig. 5c). Furthermore, such colocalized cells were not as abundant in the pemafibrate group as they were in the NMDA group (Fig. 5c).

## Discussion

Our study revealed a significant neuroprotective effect of systemic administration of pemafibrate on NMDA-induced retinal neuronal excitotoxicity. A previous study showed a neuroprotective effect of oral fenofibrate administration in a Parkinson’s disease model rat [30]. Other study demonstrated that fenofibrate treatment significantly ameliorated neuronal death in hippocampus following global cerebral ischemia/reperfusion [31]. In addition, it was shown that daily administration of fenofibrate led to neuroprotection in a transgenic mice model of amyotrophic lateral sclerosis [32]. On the other hand, some studies showed the effect of PPAR-α agonist on retinal vessels. For example, oral administration of pemafibrate has been shown to prevent pathological angiogenesis in the retina by increasing FGF21 levels in a mouse model of oxygen-induced retinopathy [33]. In a very recent study, pemafibrate was shown to ameliorate diabetes-induced retinal vascular leukostasis and leakage via thrombomodulin upregulation [34]. Taken together, it is likely that pemafibrate has a beneficial effect not only in terms of vascular permeability but also in terms of neuronal cell survival in retina.

The present study showed that vitreous administration of NMDA led to a significant elevation in the p-c-Jun protein level, and this increase was significantly prevented by systemic pemafibrate treatment. The increase in p-c-Jun 12 h after NMDA injection was consistent with our previous study showing significant increases 6 h and 12 h after NMDA administration [35]. It was shown that ET-1 increased the p-c-Jun protein level and this increase was markedly inhibited by fenofibrate in cultured cardiomyocytes [36]. It is interesting to note that fenofibrate reduced cardiac fibrosis via the inhibition of c-Jun activity [37]. There is a possibility that other signaling pathway participates in the protective effect of pemafibrate because of the discrepancy between large reduction in p-c-Jun and modest protection in RGCs. However, since we previously found that inhibition of p-c-Jun resulted in neuronal cell protection in the RGCL against NMDA injection [35], it is reasonable to speculate that the protective effect of pemafibrate is accompanied with p-c-Jun inhibition. As reported previously [35], p-c-Jun was colocalized with Thy-1 immunoreactivity, and the increased p-c-Jun was ameliorated by pemafibrate treatment, thus implicating decreased p-c-Jun in RGCs may be involved in RGC protection by pemafibrate.

It is well known that vitreous administration of NMDA causes abundant TUNEL-positive cells in the RGCL and INL [38]. The current study also confirmed the presence of abundant TUENL-positive cells in the RGCL and INL. The number of TUNEL-positive cells in the RGCL was significantly lessened by systemic administration of pemafibrate, while the reduction of TUNEL-positive cell number was not significant in the INL. One hypothesis posits that the effects of pemafibrate may vary dependent on cell types as the INL consists of the nucleus of amacrine cells, bipolar cells, horizontal cells, and Müller cells. Colocalization of TUNEL-positive cells and p-c-Jun immunopositive cells implicates the involvement of p-c-Jun in apoptosis, and these colocalized cells were decreased by pemafibrate treatment. In line with this finding, previous studies have suggested that PPAR-α agonists suppress apoptosis. For example, fenofibrate was shown to inhibit palmitate-induced myocardial apoptosis in mice [39]. Moreover, a recent study showed that daily intraperitoneal injection of fenofibric acid significantly decreased retinal apoptosis in STZ-induced diabetes [40]. Hence, long-term studies will be needed to support the idea that pemafibrate has potential for treatment of diabetic retinopathy since apoptosis is involved in the pathophysiology of this disease [41]. Furthermore, more beneficial effects of pemafibrate include usefulness for patients who have renal dysfunction because it is metabolized in the liver [42]. Other effects the pemafibrate could have are protective effects against atherosclerosis and inflammation in hyperlipidemic condition [42]. It has been reported that oral fenofibrate reduced caspase-3 immunoreactivity in a rat stroke model and that prior administration of fenofibrate resulted in a significant reduction in infarct size [43]. Therefore, it is possible that the neuroprotective effect of pemafibrate is accompanied by an anti-apoptotic effect. Further studies are needed to clarify the mechanism underlying the neuroprotection provided by pemafibrate.

## References

[1] Berger J, Moller DE (2002). The mechanisms of action of PPARs. Annu Rev Med.

[2] Staels B, Dallongeville J, Auwerx J, Schoonjans K, Leitersdorf E, Fruchart JC (1998). Mechanism of action of fibrates on lipid and lipoprotein metabolism. Circulation.

[3] Hu Y, Chen Y, Ding L, He X, Takahashi Y, Gao Y, Shen W, Cheng R, Chen Q, Qi X, Boulton ME, Ma JX (2013). Pathogenic role of diabetes-induced PPAR-α down-regulation in microvascular dysfunction. Proc Natl Acad Sci U S A.

[4] Moran E, Ding L, Wang Z, Cheng R, Chen Q, Moore R, Takahashi Y, Ma JX (2014). Protective and antioxidant effects of PPARα in the ischemic retina. Invest Ophthalmol Vis Sci.

[5] Fruchart JC (2009). Peroxisome proliferator-activated receptor-alpha (PPARalpha): at the crossroads of obesity, diabetes and cardiovascular disease. Atherosclerosis.

[6] Bougarne N, Weyers B, Desmet SJ, Deckers J, Ray DW, Staels B, De Bosscher K (2018). Molecular actions of PPARα in lipid metabolism and inflammation. Endocr Rev.

[7] Wahli W, Michalik L (2012). PPARs at the crossroads of lipid signaling and inflammation. Trends Endocrinol Metab Jul.

[8] Keech AC, Mitchell P, Summanen PA, O’Day J, Davis TM, Moffitt MS, Taskinen MR, Simes RJ, Tse D, Williamson E, Merrifield A, Laatikainen LT, d’Emden MC, Crimet DC, O’Connell RL, Colman PG, FIELD study investigators (2007). Effect of fenofibrate on the need for laser treatment for diabetic retinopathy (FIELD study): a randomised controlled trial. Lancet.

[9] Chew EY, Ambrosius WT, Howard LT, Greven CM, Johnson S, Danis RP, Davis MD, Genuth S, Domanski M, ACCORD Study Group (2007). Rationale, design, and methods of the Action to Control Cardiovascular Risk in Diabetes Eye Study (ACCORD-EYE). Am J Cardiol.

[10] Chew EY, Ambrosius WT, Davis MD, Danis RP, Gangaputra S, Greven CM, Hubbard L, Esser BA, Lovato JF, Perdue LH, Goff DC, Cushman WC, Ginsberg HN, Elam MB, Genuth S, Gerstein HC, Schubart U, Fine LJ, ACCORD Study Group, ACCORD Eye Study Group (2010). Effects of medical therapies on retinopathy progression in type 2 diabetes. N Engl J Med.

[11] Chew EY, Davis MD, Danis RP, Lovato JF, Perdue LH, Greven C, Genuth S, Goff DC, Leiter LA, Ismail-Beigi F, Ambrosius WT, Action to Control Cardiovascular Risk in Diabetes Eye Study Research Group (2014). The effects of medical management on the progression of diabetic retinopathy in persons with type 2 diabetes: the action to control cardiovascular risk in diabetes (ACCORD) eye study. Ophthalmology.

[12] Liu Q, Zhang F, Zhang X, Cheng R, Ma JX, Yi J, Li J (2018). Fenofibrate ameliorates diabetic retinopathy by modulating Nrf2 signaling and NLRP3 inflammasome activation. Mol Cell Biochem.

[13] Hennuyer N, Duplan I, Paquet C, Vanhoutte J, Woitrain E, Touche V, Colin S, Vallez E, Lestavel S, Lefebvre P, Staels B (2016). The novel selective PPARα modulator (SPPARMα) pemafibrate improves dyslipidemia, enhances reverse cholesterol transport and decreases inflammation and atherosclerosis. Atherosclerosis.

[14] Pradhan AD, Paynter NP, Everett BM, Glynn RJ, Amarenco P, Elam M, Ginsberg H, Hiatt WR, Ishibashi S, Koenig W, Nordestgaard BG, Fruchart JC, Libby P, Ridker PM (2018). Rationale and design of the Pemafibrate to reduce cardiovascular outcomes by reducing triglycerides in patients with diabetes (PROMINENT) study. Am Heart J.

[15] Ida S, Kaneko R, Murata K (2019). Efficacy and safety of pemafibrate administration in patients with dyslipidemia: a systematic review and meta-analysis. Cardiovasc Diabetol.

[16] Kawasaki M, Kambe A, Yamamoto Y, Arulmozhiraja S, Ito S, Nakagawa Y, Tokiwa H, Nakano S, Shimano H (2020). Elucidation of molecular mechanism of a selective PPARα modulator, pemafibrate, through combinational approaches of X-ray crystallography, thermodynamic analysis, and first-principle calculations. Int J Mol Sci.

[17] Oshitari T, Bikbova G, Yamamoto S (2014). Increased expression of phosphorylated c-Jun and phosphorylated c-Jun N-terminal kinase associated with neuronal cell death in diabetic and high glucose exposed rat retinas. Brain Res Bull.

[18] Wang YS, Zhou JP, Wei ZF, Tian QY, Zhou HX, Zhang YX (2007). Effect of phosphorylated c-Jun expression on COX-2 expression in the substantia nigra of MPTP mouse model of subacute Parkinson disease [in Chinese]. Nan Fang Yi Ke Da Xue Bao.

[19] Ambati J, Chalam KV, Chawla DK, D’Angio CT, Guillet EG, Rose SJ, Vanderlinde RE, Ambati BK (1997). Elevated gamma-aminobutyric acid, glutamate, and vascular endothelial growth factor levels in the vitreous of patients with proliferative diabetic retinopathy. Arch Ophthalmol.

[20] Gowda K, Zinnanti WJ, LaNoue KF (2011). The influence of diabetes on glutamate metabolism in retinas. J Neurochem.

[21] Lieth E, Barber AJ, Xu B, Dice C, Ratz MJ, Tanase D, Strother JM (1998). Glial reactivity and impaired glutamate metabolism in short-term experimental diabetic retinopathy. Penn State Retina Research Group. Diabetes.

[22] Kitaoka Y, Munemasa Y, Nakazawa T, Ueno S (2007). NMDA-induced interleukin-1beta expression is mediated by nuclear factor-kappa B p65 in the retina. Brain Res.

[23] Barber AJ, Lieth E, Khin SA, Antonetti DA, Buchanan AG, Gardner TW (1998). Neural apoptosis in the retina during experimental and human diabetes. Early onset and effect of insulin. J Clin Invest.

[24] Gastinger MJ, Singh RS, Barber AJ (2006). Loss of cholinergic and dopaminergic amacrine cells in streptozotocin-diabetic rat and Ins2Akita-diabetic mouse retinas. Invest Ophthalmol Vis Sci.

[25] Martin PM, Roon P, Van Ells TK, Ganapathy V, Smith SB (2004). Death of retinal neurons in streptozotocin-induced diabetic mice. Invest Ophthalmol Vis Sci.

[26] Park SH, Park JW, Park SJ, Kim KY, Chung JW, Chun MH, Oh SJ (2003). Apoptotic death of photoreceptors in the streptozotocin-induced diabetic rat retina. Diabetologia.

[27] Kitaoka Y, Kitaoka Y, Kwong JM, Ross-Cisneros FN, Wang J, Tsai RK, Sadun AA, Lam TT (2006). TNF-alpha-induced optic nerve degeneration and nuclear factor-kappaB p65. Invest Ophthalmol Vis Sci.

[28] Kitaoka Y, Kumai T, Isenoumi K, Kitaoka Y, Motoki M, Kobayashi S, Ueno S (2003). Neuroprotective effect of nitric oxide against NMDA-induced neurotoxicity in the rat retina is associated with tyrosine hydroxylase expression. Brain Res.

[29] Munemasa Y, Ohtani-Kaneko R, Kitaoka Y, Kuribayashi K, Isenoumi K, Kogo J, Yamashita K, Kumai T, Kobayashi S, Hirata K, Ueno S (2005). Contribution of mitogen-activated protein kinases to NMDA-induced neurotoxicity in the rat retina. Brain Res.

[30] Barbiero JK, Santiago R, Tonin FS, Boschen S, da Silva LM, Werner MF, da Cunha C, Lima MM, Vital MA (2014). PPAR-α agonist fenofibrate protects against the damaging effects of MPTP in a rat model of Parkinson’s disease. Prog Neuro-Psychopharmacol Biol Psychiatry.

[31] Xuan AG, Chen Y, Long DH, Zhang M, Ji WD, Zhang WJ, Liu JH, Hong LP, He XS, Chen WL (2015). PPARα agonist fenofibrate ameliorates learning and memory deficits in rats following global cerebral ischemia. Mol Neurobiol.

[32] Esmaeili MA, Yadav S, Gupta RK, Waggoner GR, Deloach A, Calingasan NY, Beal MF, Kiaei M (2016). Preferential PPAR-α activation reduces neuroinflammation, and blocks neurodegeneration in vivo. Hum Mol Genet.

[33] Tomita Y, Ozawa N, Miwa Y, Ishida A, Ohta M, Tsubota K, Kurihara T (2019). Pemafibrate prevents retinal pathological neovascularization by increasing FGF21 level in a murine oxygen-induced retinopathy model. Int J Mol Sci.

[34] Shiono A, Sasaki H, Sekine R, Abe Y, Matsumura Y, Inagaki T, Tanaka T, Kodama T, Aburatani H, Sakai J, Takagi H (2020). PPARα activation directly upregulates thrombomodulin in the diabetic retina. Sci Rep.

[35] Munemasa Y, Ohtani-Kaneko R, Kitaoka Y, Kumai T, Kitaoka Y, Hayashi Y, Watanabe M, Takeda H, Hirata K, Ueno S (2006). Pro-apoptotic role of c-Jun in NMDA-induced neurotoxicity in the rat retina. J Neurosci Res.

[36] Irukayama-Tomobe Y, Miyauchi T, Sakai S, Kasuya Y, Ogata T, Takanashi M, Iemitsu M, Sudo T, Goto K, Yamaguchi I (2004). Endothelin-1-induced cardiac hypertrophy is inhibited by activation of peroxisome proliferator-activated receptor-alpha partly via blockade of c-Jun NH2-terminal kinase pathway. Circulation.

[37] Ichihara S, Li P, Mise N, Suzuki Y, Izuoka K, Nakajima T, Gonzalez F, Ichihara G (2019). Ablation of aryl hydrocarbon receptor promotes angiotensin II-induced cardiac fibrosis through enhanced c-Jun/HIF-1α signaling. Arch Toxicol.

[38] Lam TT, Abler AS, Kwong JM, Tso MO (1999). N-methyl-D-aspartate (NMDA)-induced apoptosis in rat retina. Invest Ophthalmol Vis Sci.

[39] Kong JY, Rabkin SW (2004). Reduction of palmitate-induced cardiac apoptosis by fenofibrate. Mol Cell Biochem.

[40] Pearsall EA, Cheng R, Matsuzaki S, Zhou K, Ding L, Ahn B, Kinter M, Humphries KM, Quiambao AB, Farjo RA, Ma JX (2019). Neuroprotective effects of PPARα in retinopathy of type 1 diabetes. PLoS One.

[41] Li X, Zhang M, Zhou H (2014). The morphological features and mitochondrial oxidative stress mechanism of the retinal neurons apoptosis in early diabetic rats. J Diabetes Res.

[42] Yamashita S, Masuda D, Matsuzawa Y (2020). Pemafibrate, a new selective PPARα modulator: drug concept and its clinical applications for dyslipidemia and metabolic diseases. Curr Atheroscler Rep.

[43] Altintas O, Altintas MO, Aydin MS, Baran O, Antar V, Esrefoglu M, Asil T (2017). Neuroprotective effects of chronic fenofibrate treatment via modulating the immunoreactivity of cleaved caspase-3 in stroke induced by transient middle cerebral artery occlusion rat model. Turk Neurosurg.

